# Vestibular function after simultaneous bilateral cochlear implantation in adults

**DOI:** 10.3389/fneur.2023.1304927

**Published:** 2023-11-03

**Authors:** Jun Yokoi, Takeshi Fujita, Natsumi Uehara, Shinobu Iwaki, Akinobu Kakigi, Ken-ichi Nibu

**Affiliations:** Department of Otolaryngology - Head and Neck Surgery, Kobe University Graduate School of Medicine, Kobe, Japan

**Keywords:** cochlear implant, bilateral simultaneous cochlear implantation, vestibular function, VEMP, static stabilometry

## Abstract

**Introduction:**

Binaural hearing enhances speech intelligibility, source localization, and speech comprehension in noisy environments. Although bilateral cochlear implantation (CI) offers several benefits, concerns arise regarding the risk of bilateral postoperative vestibular dysfunction with simultaneous CI. This study aimed to longitudinally evaluate changes in vestibular function in adult patients who underwent simultaneous bilateral CI using minimally invasive electrodes and surgical techniques.

**Methods:**

A retrospective review was conducted on 10 patients who underwent simultaneous bilateral CI at our hospital. Vertigo symptoms and vestibular function test results were examined preoperatively, 1–6 months postoperatively, and 1 year postoperatively. Nystagmus tests, caloric reflex tests, vestibular evoked myogenic potentials (VEMP) measurements, and static stabilometry were performed as vestibular function tests.

**Results:**

Although an initial transient decline in vestibular function was observed, no significant long-term decline was observed in the caloric reflex test, ocular VEMP (oVEMP), or cervical VEMP (cVEMP). Moreover, regardless of the presence or absence of abnormalities in caloric reflex, oVEMP, or cVEMP, no significant deterioration was detected in the static stabilometer test. While two patients reported preoperative dizziness, all patients were symptom-free 1 year postoperatively.

**Discussion:**

The findings suggest that using current minimally invasive electrodes and surgical techniques in simultaneous bilateral CI leads to temporary vestibular function decline postoperatively. However, most patients experience a recovery in function over time, highlighting the potential safety and efficacy of the procedure. Simultaneous bilateral CI surgery is viable, depending on the patient’s auditory needs and burden.

## 1. Introduction

Binaural hearing provides advantages over unilateral hearing in terms of speech intelligibility, source localization, and understanding of speech in noise, which are explained by the head shadow, squelch, summation, and localization effects in binaural hearing (1). Bilateral cochlear implantation (CI) is superior in noise audibility, directional localization (2), and other functions, as well as in language development in children (3). Prof. Müller and his colleagues in Würzburg, Germany, were the first to perform a bilateral CI on an adult in 1996 and on a pediatric patient in 1998. Following these milestones, research groups from around the world have carried out numerous clinical studies (2). Today, bilateral CI is recognized as a standard treatment in several countries, with costs often covered by their health care systems. Compared to sequential surgery, simultaneous bilateral CI is thought to produce binaural effects at an earlier stage and facilitate the integration of the bilateral auditory cortex at the central level. An additional patient benefit is the need for a single hospitalization. However, CIs have been associated with dizziness and vestibular dysfunction (4–8), raising concerns regarding the risk of bilateral postoperative vestibular dysfunction with simultaneous CI.

Recent advances in electrode design for cochlear implants and innovative surgical techniques have aimed to preserve inner ear function (8–12). Consequently, minimally invasive surgeries have become increasingly prevalent. While numerous reports and meta-analyses (13, 14) have examined vestibular function in patients who have undergone CI, only one study has assessed vestibular function in patients who have undergone simultaneous bilateral CI (15). This report compared vestibular function preoperatively with that at 4 months postoperatively and found no reduction in the dizziness handicap inventory (DHI), utricle, and semicircular canal function; however, saccule function decreased. To date, no study has conducted long-term longitudinal measurements in this patient group.

In this study, we investigated changes in vestibular function over time using the caloric reflex test, vestibular evoked myogenic potential (VEMP), and static stabilometry in adult patients who had undergone simultaneous bilateral CI using the latest minimally invasive electrodes and surgical techniques that are currently mainstream in the field.

## 2. Materials and methods

### 2.1. Participants

A retrospective review was conducted on 10 patients who underwent simultaneous bilateral CI at our hospital between April 2018 and April 2022. Vertigo symptoms and vestibular function test results were examined preoperatively, 1–6 months postoperatively, and 1 year postoperatively. This study was approved by the Institutional Review Board of Kobe University Hospital (B210006).

Nystagmus tests, caloric reflex tests, VEMP measurements, and static stabilometry were performed as vestibular function tests. The caloric reflex test and VEMP were omitted in patients who underwent preoperative canal wall-down mastoidectomy or external auditory canal closure.

### 2.2. Surgical procedure

The round window niche was identified via posterior tympanotomy following canal wall-up mastoidectomy. After removing the bony overhang of the niche, a small incision was made in the round window membrane using a micro ear pick. The electrode was then slowly inserted. Steroids were administered to all patients. The dexamethasone protocol was as follows: 6.6 mg administered intravenously at the beginning of surgery, 1.65 mg applied around the round window after the opening of the posterior tympanic chamber, 6.6 mg administered intravenously on postoperative days 1 and 2, and 3.3 mg administered intravenously on postoperative days 3 and 4.

### 2.3. Electrode angular insertion depth

Angular insertion depth was measured on postoperative CT-scan, using the measurement method as advised by the Consensus Panel in Verbist et al. (16).

### 2.4. Symptoms

Data on the presence or absence of pre-and postoperative vertigo symptoms were obtained from the medical records.

### 2.5. Nystagmus tests

The nystagmus tests included gaze, spontaneous, head, and head-tilt tests using an infrared charge-coupled device (CCD) camera (Newopto, Kanagawa, Japan).

### 2.6. Caloric reflex test

For the caloric reflex test, the maximum slow-phase velocity was measured using electronystagmography (Rion, Tokyo, Japan) with a simple cold caloric test method (5 mL of cold water at 20°C for 20 s) (17). If nystagmus was not elicited, it was considered a lack of response. A maximum slow-phase velocity < 10°/s was considered to represent hypofunction (17).

### 2.7. VEMP

Both ocular VEMP (oVEMP) and cervical VEMP (cVEMP) were used for VEMP testing. The stimulus tone used was a 105 dBnHL 500 Hz short-tone burst for air conduction stimulation. The electrographic signal was recorded using a Neuropack evoked potential recorder (Nihon Kohden, Tokyo, Japan). No response was recorded when no reproducible waveform was observed, and a normal response when a reproducible waveform was observed. The n1-p1 interpulse amplitude was measured using oVEMP, and the p13-n23 interpulse amplitude was measured using cVEMP.

### 2.8. Static stabilometer test

In the static stabilometer test, the participants were instructed to stand for 1 min for each eye opening and closing, and the area, speed, and Romberg ratio were measured in the CI-on and CI-off modes using a stabilometer (Anima, Tokyo, Japan).

### 2.9. Statistics

We compared the results at 1, 3, and 6 months, and 1 year postoperatively using a one-way ANOVA followed by Dunnett’s multiple comparisons test, with the preoperative period as a control. A *t*-test was used to assess the differences between CI-on and-off modes for each parameter in the static stabilometer test. All statistical analyses were performed using GraphPad Prism 9 software (GraphPad Software, San Diego, CA, USA). All tests were conducted at a significance level of *p* < 0.05.

## 3. Results

### 3.1. Patient background

The characteristics of the 10 patients who underwent vestibular function tests preoperatively to 1 year postoperatively are shown in Table 1. The median patient age was 65 (23–83) years. Case 1 with bilateral cholesteatoma otitis media had undergone bilateral tympanoplasty (combined with right external auditory canal closure) 1 year before CI, and intraoperative findings on the right showed a semicircular canal fistula due to cholesteatoma. The FLEXSOFT (MED-EL, Innsbruck, Austria) electrode was most commonly used, with bilateral insertions observed in 5 patients. Almost all were inserted using the round window approach (RWA) or extended RWA; however, one patient with autoimmune sensorineural hearing loss had undergone insertion via cochleostomy because it was difficult to insert the electrodes using RWA on the right side. The mean electrode angular insertion depth was 526.6° (range 393.3–579.5). All patients’ pre-and postoperative hearing level is shown in Supplemental Digital Material 1.

**Table 1:** Characteristics of patients who underwent simultaneous bilateral cochlear implantation.

Case	Age	Sex	Electrodes, both sides (company)	Insertion method (side)	Electrode angular insertion depth (side)	Cause of hearing loss
1	70	F	FLEXSOFT (MED-EL)	RWA (both)	536° (right), 547° (left)	Cholesteatoma
2	38	M	FLEXSOFT (MED-EL)	RWA (both)	NR	Unknown
3	63	M	FLEXSOFT (MED-EL)	RWA (both)	NR	Unknown
4	83	F	FLEXSOFT (MED-EL)	Cochleostomy (right), eRWA (left)	522° (right), 559° (left)	Autoimmune sensorineural hearing loss
5	75	M	FLEXSOFT (MED-EL)	RWA (both)	530° (right), 555° (left)	Unknown
6	23	F	Standard (MED-EL)	eRWA (both)	579° (right), 536° (left)	Meningitis
7	70	F	Standard (MED-EL)	RWA (right), eRWA (left)	NR	Unknown
8	66	F	FLEX28 (MED-EL)	RWA (both)	567° (right), 569° (left)	Pendred Syndrome
9	39	F	FLEX24 (MED-EL)	RWA (both)	519° (right), 506° (left)	*KCNQ4* gene mutation
10	55	F	CI632 (Cochlear)	RWA (right), eRWA (left)	455° (right), 393° (left)	Mitochondrial hearing loss (m.1555A > G)

M, Male; F, Female; RWA, Round Window Approach; eRWA, extended Round Window Approach; NR, not recorded in the medical record.

### 3.2. Symptoms and nystagmus

Table 2 presents the data on the presence or absence of dizziness and nystagmus from the preoperative period to 1 year postoperatively in all patients. Two patients had preoperative dizziness, but 1 year postoperatively, dizziness had resolved in all patients.

**Table 2:** Dizziness symptoms and nystagmus before and after cochlear implantation (CI).

Case	before CI		A day after CI		1 month after CI		3 months after CI		6 months after CI		1 year after CI	
	Symptoms	Nystagmus	Symptoms	Nystagmus	Symptoms	Nystagmus	Symptoms	Nystagmus	Symptoms	Nystagmus	Symptoms	Nystagmus
1	+	−	−	−	+	−	NR	NR	NR	NR	−	−
2	−	−	−	NR	−	−	−	−	−	−	−	−
3	−	−	+	−	−	−	−	−	−	−	−	−
4	+	−	NR	NR	+	−	−	−	−	−	−	−
5	−	−	−	−	−	−	−	−	−	−	−	−
6	−	−	−	−	−	−	NR	NR	−	−	−	−
7	−	−	+	+	−	−	−	−	−	−	−	−
8	−	−	−	−	−	−	−	−	−	−	−	−
9	−	−	−	−	−	−	−	−	−	−	−	−
10	−	−	−	−	−	−	−	−	−	−	−	−

Abnormal findings are shown in gray. +: present, −: absent, NR, not recorded in the medical record.

### 3.3. Caloric reflex test

**Figure 1:**
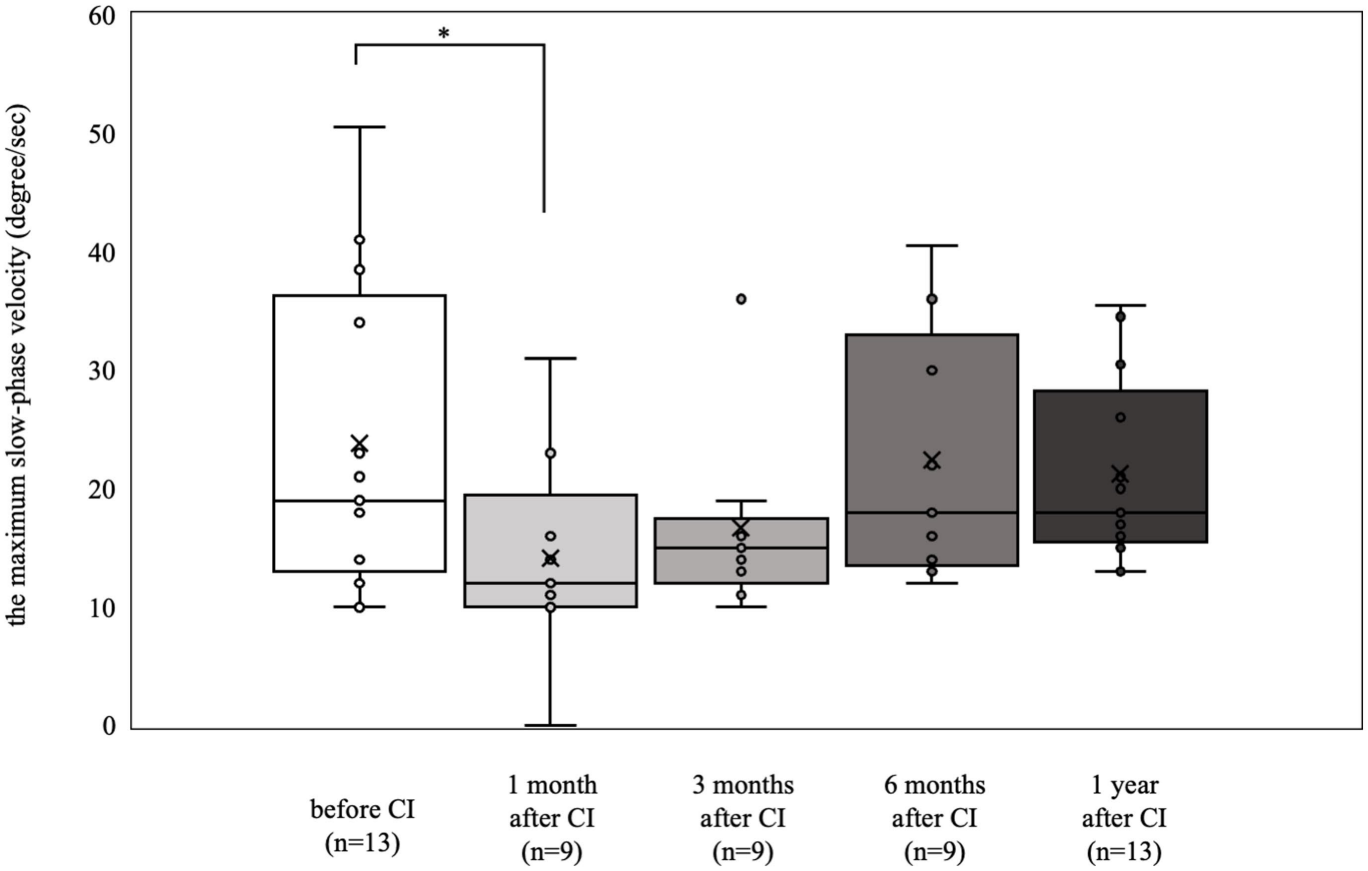
Caloric reflex test results over time. Among the 7 patients (13 ears) who exhibited a preoperative response with a maximum slow-phase velocity of 10°/sec or more, there was a notable decrease in response at 1 month postoperatively (*p < 0.05). At 3 months, 6 months, and 1 year postoperatively, the results were not significantly different from the preoperative measurements.

The results of the caloric reflex tests are shown in Figure 1. Nine patients (18 ears) were tested preoperatively, and nystagmus was not induced in 3 of them (5 ears). Seven patients (13 ears) who showed a preoperative response with a maximum slow phase velocity of 10°/s or greater (mean 23.8°/s) showed a decrease in response 1 month postoperatively (mean 14.1°/s, *p* < 0.05). However, from 3 months postoperatively to 1 year postoperatively, there was no significant deterioration compared with the preoperative value (preoperative mean 23.8°/s, 3 months postoperative mean 16.7°/s, 6 months postoperative mean 22.4°/s, 1 year postoperative mean 21.3°/s).

### 3.4. VEMP

**Figure 2:**
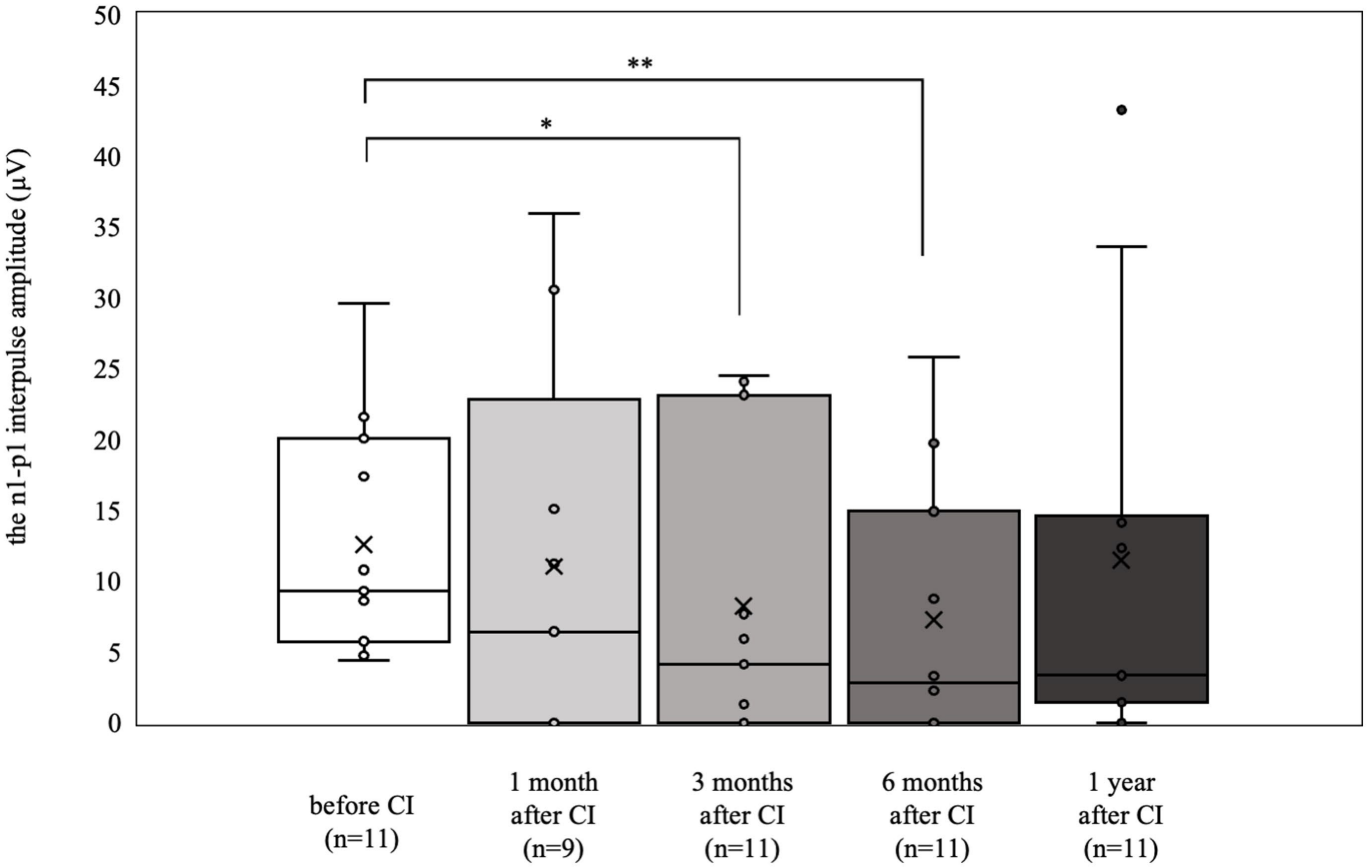
oVEMP results over time. Of the 6 patients (11 ears) who showed normal results preoperatively, significant deteriorations were observed at 3 and 6 months postoperatively (*p < 0.05, **p < 0.01). By contrast, at 1 year postoperatively, the results were not significantly different from the preoperative values.

The oVEMP results are shown in Figure 2. Nine patients (18 ears) were tested preoperatively, and 4 (7 ears) had a reduced or absent response compared with the contralateral side. The 6 patients (11 ears) who had normal results preoperatively (mean 12.6 μV) were significantly worse at 3 and 6 months postoperatively (3 months postoperative mean 8.3 μV, *p* < 0.05, 6 months postoperative mean 7.3 μV, *p* < 0.01). However, there was no significant difference at 1 year postoperatively (mean 11.5 μV).

**Figure 3:**
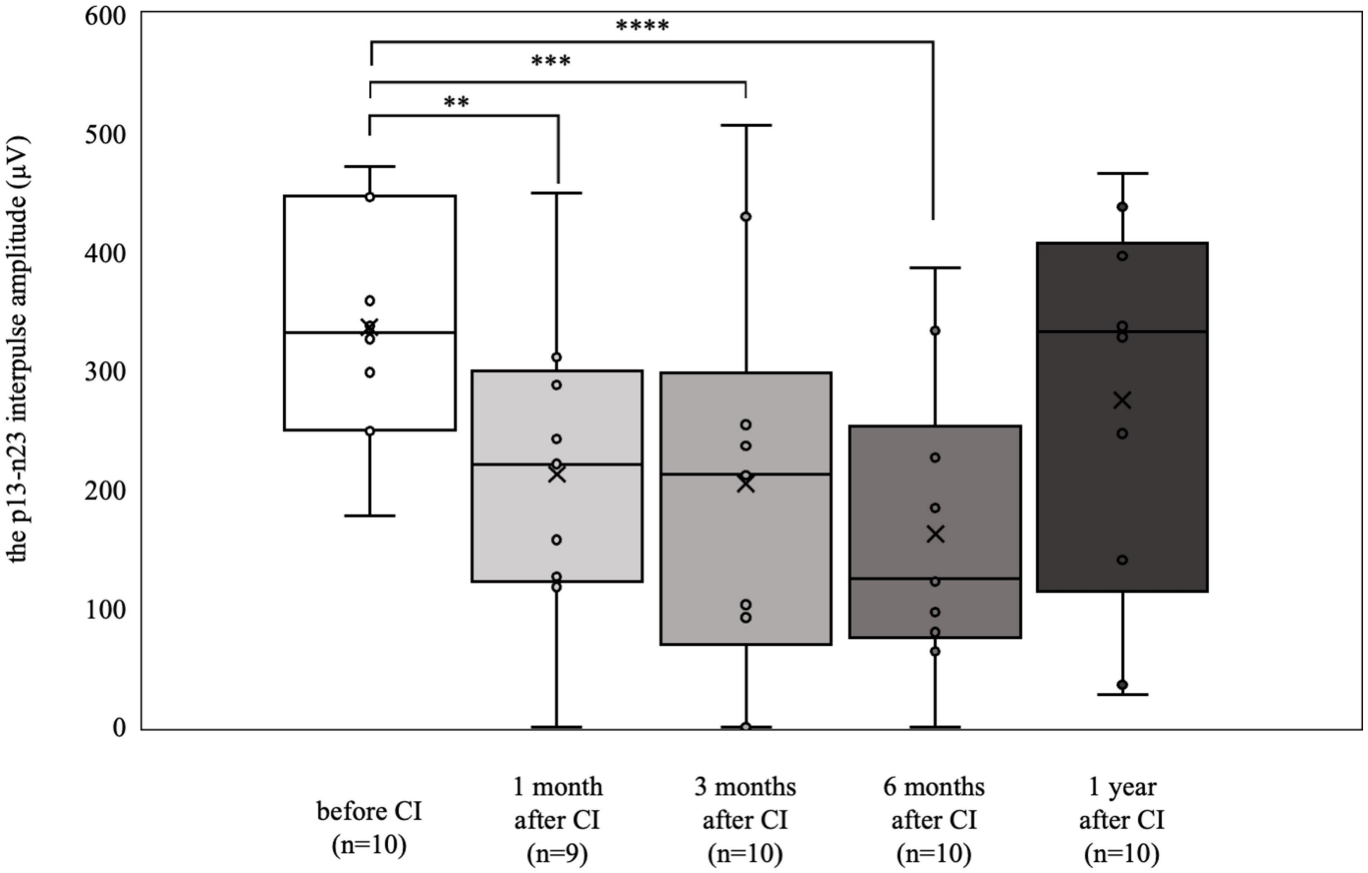
cVEMP results over time. Among the 6 patients (10 ears) who showed normal results preoperatively, significant deteriorations were observed at 1, 3, and 6 months postoperatively (**p < 0.01, ***p < 0.001, ****p < 0.0001). By 1 year postoperatively, the results were not significantly different from the preoperative values.

The cVEMP results are shown in Figure 3. Eight patients (15 ears) were preoperatively examined. Only 1 patient had difficulty in maintaining cervical elevation and was therefore omitted from the examination. Four patients (5 ears) showed a reduced or absent response compared with the contralateral side. The 6 patients (10 ears) who had normal results preoperatively (mean 337.0 μV) had significantly decreased responses at 1, 3, and 6 months postoperatively (1 month postoperative mean: 212.9 μV, *p* < 0.01; 3 months postoperative mean: 204.9 μV, *p* < 0.001; 6 months postoperative mean: 162.4 μV, *p* < 0.0001). Conversely, at 1 year postoperatively, there was no significant difference from results obtained preoperatively (mean 275.7 μV).

### 3.5. Static stabilometer test

**Figure 4:**
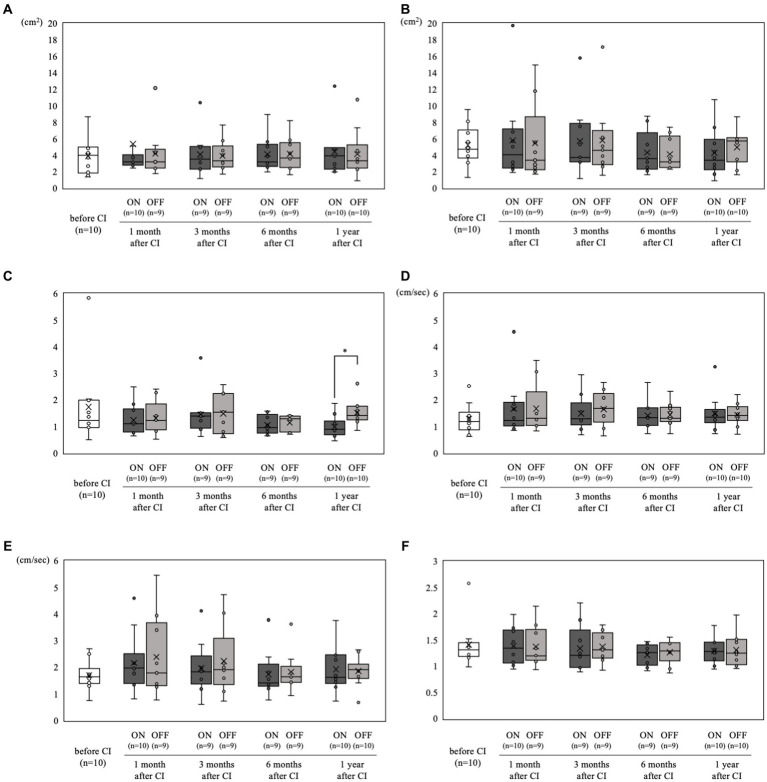
Static stabilometer test results over time. **(A)** Area with open eyes. **(B)** Area with closed eyes. **(C)** Romberg ratio of area. **(D)** Velocity with open eyes. **(E)** Velocity with closed eyes. **(F)** Romberg ratio of velocity. Comparisons between pre-and postoperative values for each parameter showed no significant differences. Moreover, when comparing on-and off-mode at each time point in the static stabilometer test, **(C)** the on-mode demonstrated a significantly better Romberg ratio of area at 1 year postoperatively (*p < 0.05).

The static stabilometer test results are shown in Figure 4. All tests were performed in all patients. Only in case 1, the static stabilometer test was not performed at 3 and 6 months postoperatively, nor was it measured in off mode at 1 month postoperatively. There were no significant differences in any of the parameters between the preoperative and postoperative results. In addition, when comparing the on-and off-mode at each stage of the static stabilometer test, the on-mode was significantly better only in the Romberg ratio of area (Figure 4C) at 1 year postoperatively (mean 0.98 vs. 1.52, *p* < 0.05).

## 4. Discussion

In this study, we conducted 1 year of postoperative follow-up of patients who underwent simultaneous bilateral CI. Initially, a transient worsening of vestibular function was observed; however, overall, there was no significant long-term deterioration in caloric reflex, oVEMP, or cVEMP. Moreover, regardless of the presence or absence of abnormalities in caloric reflex, oVEMP, or cVEMP, no significant deterioration was detected in the static stabilometer test. This test series integrates vestibular function with other senses, such as vision and proprioception, in postural maintenance. No new cases of subjective vertigo have been reported.

As the vestibular organ is close to the cochlea, it has been suggested that the insertion of CI electrodes into the cochlea may damage the vestibular organ and cause dizziness (18). There have been many reports of dizziness and vestibular disturbances after CI, with the reported frequency varying from 2.9 to 74% (4–7). The insertion of electrodes into the cochlea can cause two types of changes: (1) immediate changes due to damage to the ossicular lacunar plate, basilar membrane, and spiral ligament and (2) delayed changes due to tissue responses to the electrodes, such as inflammation, fibrosis, and osteogenesis (19). Many studies have conducted histopathological analyses of the inner ear after CI electrode insertion, and it has been noted that saccular lesions (20–22), exocrine lymph loss (20, 22), acute labyrinthitis (22), foreign body-reactive otitis media (20, 21), and ductus reunion obstruction (20, 21) occur after CI. The detailed mechanism of vestibular dysfunction after CI is unknown; however, Dagkiran et al. cited trauma caused by electrode insertion, labyrinthitis caused by a foreign body reaction, endolymphatic edema, and postoperative perilymphatic fistula (23) as possible mechanisms. Buchman suggested that electrical stimulation from implants can cause pathological changes in the inner ear and vestibular dysfunction, owing to false sensory input from electrical stimulation (24).

Regarding the function of the semicircular canal and otolith in the short term up to 6 months postoperatively, the response was significantly worse at 1 month postoperatively for the caloric test, 3 and 6 months postoperatively for oVEMP, and 1, 3 and 6 months postoperatively for cVEMP. In contrast, the long-term results at 1 year postoperatively showed no significant worsening of vestibular function in any of the tests compared with the preoperative results. The short-term worsening of the tests may be due to the impaired acoustic chain caused by postoperative changes, such as fluid in the tympanic cavity, decreased heat conduction efficiency to the lateral semicircular canal, and transient vestibular disturbances caused by surgical invasion. Residual hearing loss after CI may be due to delayed progressive hearing loss caused by the immune response and fibrosis several months to years after surgery^22^; a decrease in the number of synapses connecting hair cells to the spiral ganglion following electrical stimulation has also been suggested (25). The same may be true for vestibular function; however, delayed vestibular dysfunction was not evident in this study, possibly because of the greater distance between the vestibule and the electrode compared to the cochlea. Minimally invasive techniques and innovations, such as RWA (9, 26, 27), use of non-traumatic electrodes (10, 11, 27), and steroid administration, (12, 27, 28) have been reported to preserve residual hearing and vestibular organs. In addition, histopathological evaluation of human temporal bone with CI showed that cochleostomy was significantly associated with scala vestibule fibrosis and endolymphatic hydrops, while RWA was associated with no fibrosis and no hydrops (29). Nordfalk et al. found that postoperative loss of vestibular function did not correlate with angular insertion depth when RWA and steroids were used (30).” In our study, despite half of the patients receiving the industry’s longest electrode, FLEXSOFT, there was no apparent delayed vestibular dysfunction. These minimally invasive techniques and devices may have reduced the delayed changes observed in this study.

Regardless of the short-term worsening in these tests, there was no significant worsening of any parameter in the static stabilometer tests. There have been other reports of a lack of significant worsening after CI using a static stabilometer (24, 31). Although the meta-analysis by Ibrahim et al. was not performed on the static stabilometer test owing to the small number of included studies, the collected studies found few significant differences pre-and postoperatively (13). In the present study, the long-term results of the static stabilometer test showed no significant postoperative deterioration with the cochlear implant in on-or off-mode, regardless of the presence or absence of abnormalities in other vestibular function tests, such as the VEMP or caloric reflex tests. It is also possible that social activities, which were suppressed due to severe hearing loss, increased after CI, leading to increased muscle strength and a more stable posture.

In addition, the Romberg ratio of the area in on-mode was significantly better than that in off-mode at 1 year postoperatively. There have also been several reports of greater postural stability when the cochlear implant was turned on, with the recovery of auditory information and electrical stimulation of the vestibule suggested as reasons (32, 33). Miwa et al. found marked improvement in the static stabilometer test and cVEMP with cochlear implants on compared to those with cochlear implants off, and concluded that this may be due to the electrical stimulation of the cochlear implants that induced cervical vestibulospinal reflexes (32).

Long-term results at 1 year postoperatively showed that none of the patients had symptoms of vertigo. In a meta-analysis of the association between CI and vestibular function, Ibrahim et al. reported no increase in the DHI score in 84.4% of the postoperative patients (13). In a meta-analysis, Hänsel et al. reported that 9.3% of the patients experienced dizziness after CI (34). We thought that simultaneous bilateral CI might potentially put patients at risk of bilateral vestibular dysfunction and result in a higher frequency of postoperative dizziness than unilateral CI; however, the frequency of postoperative dizziness in the present study was comparable or even better than that in other reports. Considering that none of the patients, including the two patients who had preoperative dizziness, complained of dizziness at 1 year postoperatively, and that there was no worsening of the results of the static stabilometer test, we believe that minimally invasive surgery can avoid equilibrium dysfunction that may lead to a decline in postoperative activities of daily living (ADL), even with simultaneous bilateral CI.

This study had some limitations. First, this was a small case series. Second, the median age of the study population was 65 years; therefore, it is possible that the rate of postoperative vestibular dysfunction was higher considering patient age. Third, caloric reflex tests primarily reflect unilateral semicircular canal function, oVEMP reflects unilateral utricle function, and cVEMP reflects unilateral saccular function. Only the static stabilometer test truly reflects bilateral vestibular function and other sensory sensations (visual and deep senses). Fourth, tests to measure the degree of subjective dizziness, such as the DHI, should be added in addition to objective tests of vestibular function.

In conclusion, simultaneous bilateral CI leads to a temporary, but not prolonged, reduction of vestibular function, even though half of our cases implanted with the industry’s longest electrode, FLEXSOFT. Simultaneous bilateral CI surgery is therefore viable option depending on the patient’s auditory needs and burden.

## Data availability statement

The original contributions presented in the study are included in the article/Supplementary material, further inquiries can be directed to the corresponding author.

## Ethics statement

The studies involving humans were approved by Review Board of Kobe University Hospital. The studies were conducted in accordance with the local legislation and institutional requirements. The participants provided their written informed consent to participate in this study.

## Author contributions

JY: Writing – original draft, Writing – review & editing, Conceptualization, Data curation, Methodology. TF: Funding acquisition, Supervision, Writing – original draft, Writing – review & editing. NU: Data curation, Supervision, Writing – review & editing. SI: Data curation, Writing – review & editing. AK: Conceptualization, Supervision, Writing – review & editing. K-iN: Supervision, Writing – review & editing.
