# Supplementary material for: Vestibular function after simultaneous bilateral cochlear implantation in adults
Source: Front Neurol. 2023 Nov 3;14:1304927. doi: 10.3389/fneur.2023.1304927 (PMC10657651; doi:10.3389/fneur.2023.1304927)
Supplement: Supplementary file 1 [file Table_1.DOCX]

Supplemental Digital Material 1.

Results of mean hearing level.

| **Case** | **Preoperative PTA (dB)** | | **Post 1Y-PTA (dB)** | | **Post 1Y – free field CI aided threshold (dB)** | | |
| --- | --- | --- | --- | --- | --- | --- | --- |
|  | Right | Left | Right | Left | Right | Left | Bilateral |
| 1 | 110 | 111.3 | SO | SO | 30 | 33.3 | 33.3 |
| 2 | 106.7 | 103.3 | 113.3 | 96.7 | 35 | 35 | 33.3 |
| 3 | 103.3 | 108.8 | SO | SO | 33.3 | 35 | 36.7 |
| 4 | 100 | SO | SO | SO | 33.3 | 28.3 | 31.7 |
| 5 | 93.3 | SO | SO | SO | 41.7 | 38.3 | 33.3 |
| 6 | SO | 101.7 | SO | SO | 30 | 30 | 28.3 |
| 7 | SO | 108.3 | SO | SO | 45 | 40 | 40 |
| 8 | 105 | 85 | 106.7 | 103.3 | 53.3 | 40 | 45 |
| 9 | 51.7 | 48.3 | 91.7 | 93.3 | 33.3 | 30 | 21.7 |
| 10 | 73.3 | 81.7 | 105 | 106.7 | 28.3 | 26.7 | 25 |

The mean hearing level was calculated by dividing the sum of the 500 Hz, 1000 Hz, and 2000 Hz hearing thresholds by three.

PTA: pure tone audiometry, Post 1Y: postoperative 1 year, SO: scaled out.
